# Demographic and clinical data of patients with spinal epidural angiolipomas

**DOI:** 10.1038/s41598-024-67584-8

**Published:** 2024-07-29

**Authors:** Taikun Lu, Hao Wang, Yang Liu, Xiaowei Qin, Yongliang Teng, Yubo Wang

**Affiliations:** 1 Department of Neurosurgery, First Hospital of Jilin University, 71 Xinmin Street, Changchun, 130021 Jilin People’s Republic of China; 2 Department of Neurology, First Hospital of Jilin University, Changchun, Jilin People’s Republic of China; 3 Department of Radiology, First Hospital of Jilin University, Changchun, Jilin People’s Republic of China; 4 Department of Pathology, First Hospital of Jilin University, Changchun, Jilin People’s Republic of China

**Keywords:** Spinal cord tumor, Epidural tumor, Angiolipomas, SEER program, Epidemiology, Neurology, Oncology

## Abstract

Spinal epidural angiolipomas are rare, benign, mesenchymal tumors. It remains unclear whether spinal epidural angiolipomas are genuinely rare or merely underreported. Herein, we assessed the demographic and clinical characteristics of patients with spinal epidural angiolipoma. We collected data from patients with spinal epidural angiolipoma from three sources. First, we retrospectively analyzed data from patients diagnosed with spinal epidural angiolipoma in our hospital between January 1, 2014, and December 31, 2023. Second, we performed a literature review of studies retrieved from PubMed. Third, we retrieved detailed data of patients with spinal angiolipoma from the Surveillance, Epidemiology, and End Results (SEER) database. We conducted a descriptive analysis to investigate the demographic and clinical characteristics of patients with spinal epidural angiolipoma. At our institution, three patients were diagnosed with spinal epidural angiolipoma. Additionally, we identified 116 patients from the literature review and 15 patients from the SEER database. We reviewed the treatment history and imaging features of the three patients from our institution. The descriptive analysis of the data collected from the literature review was consistent with previous reports. For example, 63.0% of lesions were located at the thoracic level. 31.9% of these lesions involved two vertebral bodies, while 75.6% involved 2–4 vertebral bodies. The most common symptoms experienced by patients were back pain, paraparesis, and numbness in the legs. Surgery was the primary treatment option for most patients, and complete tumor resection was achieved in the majority of patients. The male:female ratio was 1:1.4, the median age at diagnosis for the patients from the literature was 49 years old, and the median follow-up was 24 months. Notably, most of the reports came from Asia and there were few reports from Africa. The findings from the SEER database indicated a male:female ratio of 2:1. The peak incidence, which is typically reported in the fifth decade of life, was not observed. We presented three cases of spinal epidural angiolipoma and supplemented our findings with a literature review and population-based analysis according to the SEER database for the United States population. We believe that our research will enhance clinicians’ comprehension of this uncommon tumor.

## Introduction

Angiolipomas are benign mesenchymal tumors. These tumors are classified as lipoma variants, and they display notable vascularity. The vessels typically exhibit a capillary morphology and are most visible in the peripheral regions of the tumor. Moreover, these vessels often house scattered fibrin thrombi^1^. Angiolipomas of the central nervous system (CNS) mostly occur in the spinal canal, and almost all of these tumors are epidural^2^. Spinal epidural angiolipomas are exceedingly rare, accounting for just 0.14–1.2% of all spinal tumors and approximately 2–3% of extradural spinal tumors^3^. It remains unclear whether this tumor is genuinely rare or if it is merely underreported^4^. In the present study, we investigated the demographic and clinical characteristics of patients with spinal epidural angiolipoma with the aim of improving clinicians’ knowledge of this rare tumor and providing guidance for future research.

## Method

This study was approved by the Medical Research Ethics Committee of our institution. The informed consents were obtained from the patients from our institution and the research was performed in accordance with the Declaration of Helsinki. We collected data from patients with spinal epidural angiolipoma from three sources. First, we retrospectively analyzed demographic and clinical data from patients diagnosed with spinal epidural angiolipoma in our hospital between January 1, 2014, and December 31, 2023. Second, we searched PubMed for English-language reports of patients with spinal epidural angiolipoma. The search strategy was as follows: ((English [Language]) AND (((spinal cord tumor[MeSH Terms]) OR (spinal cord tumors[MeSH Terms])) AND (Angiolipoma[Title/Abstract]))) OR ((english[Language]) AND ((epidural[Title/Abstract]) AND ((Spinal[Title/Abstract]) AND (angiolipoma[Title/Abstract])))). The exclusion criteria were as follows: (1) nonhuman or non-English publications, (2) unknown age at diagnosis, and (3) unknown follow-up. Third, we retrieved detailed data from patients with spinal epidural angiolipomas from the Surveillance, Epidemiology, and End Results (SEER) program of the National Cancer Institute (NCI). We could not definitively determine whether the tumor location was extradural based on the variables in the SEER database. However, almost all spinal cord angiolipomas are known to be located extradurally; therefore, we searched for all spinal cord angiolipomas. Patients with spinal cord angiolipomas were identified by an International Classification of Diseases for Oncology 3 (ICD-O-3) code of 8861/0 located at the primary site of C70.1, C72.0, or C72.1. The research period was set as 2000–2020. Patients with an unknown age at diagnosis were excluded. The data were obtained by using the SEER*Stat 8.4.3 program. The latest database version, Incidence—SEER Research Limited-Field Data, 22 Registries, was utilized^5^. We conducted a comprehensive review of the clinical characteristics and treatment of patients from both our institution and those described in the studies retrieved from PubMed. Subsequently, a descriptive analysis was performed to illustrate the demographic characteristics of the patients from the SEER database and patients from published studies (including the patients from our institution).

## Results

### Cases presentation

In the past decade, three patients with spinal epidural angiolipomas have been treated at our department. All of the three cases were diagnosed as non-infiltrating spinal epidural angiolipomas.

#### Case 1

**Figure 1:**
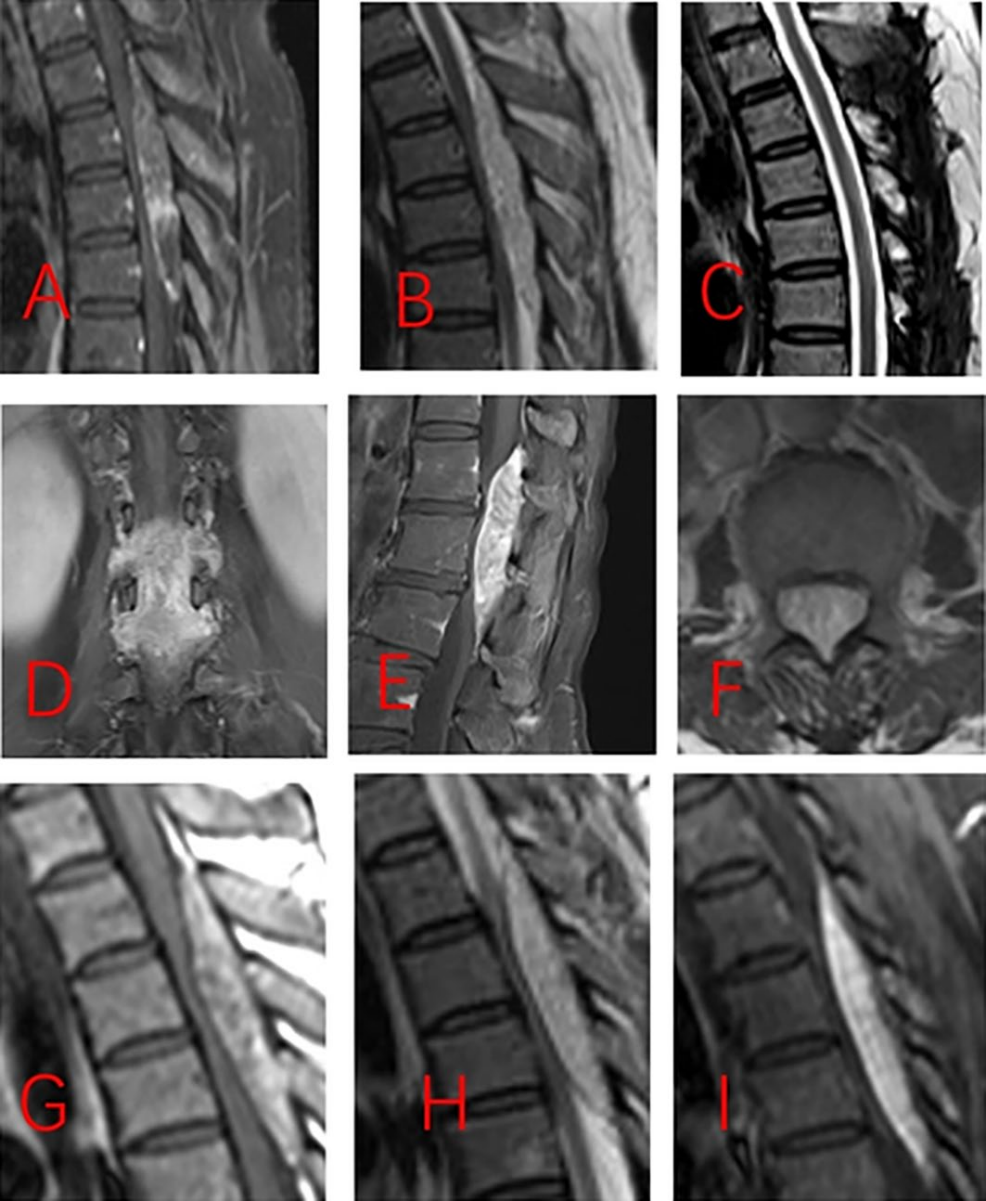
MRI of the three patients from our institution. (**A**) and (**B**): First patient’s preoperative T1WI and T2WI images. **C**: First patient’s postoperative T2WI images. (**D**), (**E**), and (**F**): The second patient’s preoperative T1 enhancement coronal, sagittal, and axial images. (**G**), (**H**) and (**I**): The third patient’s preoperative T1WI, T2WI and T1 enhancement images.

**Figure 2:**
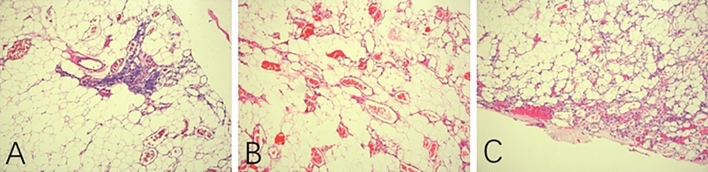
The histological images of the three patients from our institution (Hematoxylin and Eosin staining, X100). (**A**): The first patient, (**B**): The second patient, (**C**): The third patient.

The first patient was a 52-year-old female who presented with bilateral lower limb numbness for 6 months and was found to have a mass at the T4–T6 level on magnetic resonance imaging (MRI). The mass paralleled the spinal canal’s long axis located in the posterior epidural space and had a spindle or willow leaf shape, causing compression and displacement of the spinal cord (Fig. 1A, B. The mass was predominantly hyperintense on non-contrast T1-weighted imaging (T1WI) and T2-weighted imaging (T2WI) and showed inhomogeneous hyperintense enhancement. Physical examination revealed weakness in both lower extremities (Medical Research Council, MRC 4/5), with all other assessments appearing normal. The tumor was completely removed with a T4–T6 laminoplasty, and the diagnosis of angiolipoma was confirmed by histopathological analysis (Fig. 2A). The follow-ups were uneventful, and she recovered well and returned to normal life. The follow-up MRI 5 months after surgery showed no residual or recurrence of the tumor (Fig. 1C).

#### Case 2

The second patient was a 49-year-old female who presented with intermittent pain in both lower limbs for 6 years and was found to have a mass at the L1–L3 level on MRI (Fig. 1D–F). The presentation of the mass on the MRI scan was similar to that seen in the first patient. The physical examination was normal. The tumor was completely removed with a L1–L3 laminoplasty, and the diagnosis of angiolipoma was confirmed by histopathological analysis (Fig. 2B). The follow-ups were uneventful.

#### Case 3

The third patient was a 47-year-old female who presented with bilateral lower limb numbness and weakness for 1 year and was found to have a mass at the T2-T5 level on MRI (Fig. 1G–I). The mass was predominantly hyperintense on T1WI (Fig. 1G) and T2WI (Fig. 1H), and showed inhomogeneous hyperintense enhancement (Fig. 1I). Physical examination revealed weakness in both lower extremities (MRC 4/5) and increased muscle tone. Both knee and Achilles tendon reflexes were hyperactive, the Babinski sign was positive bilaterally, and both knee and ankle clonus tests were positive. The tumor was completely removed with a T2–T5 laminoplasty, and the diagnosis of angiolipoma was confirmed by histopathological analysis (Fig. 2C). The follow-ups were uneventful, and she recovered well and returned to normal life.

### Literature review

**Figure 3:**
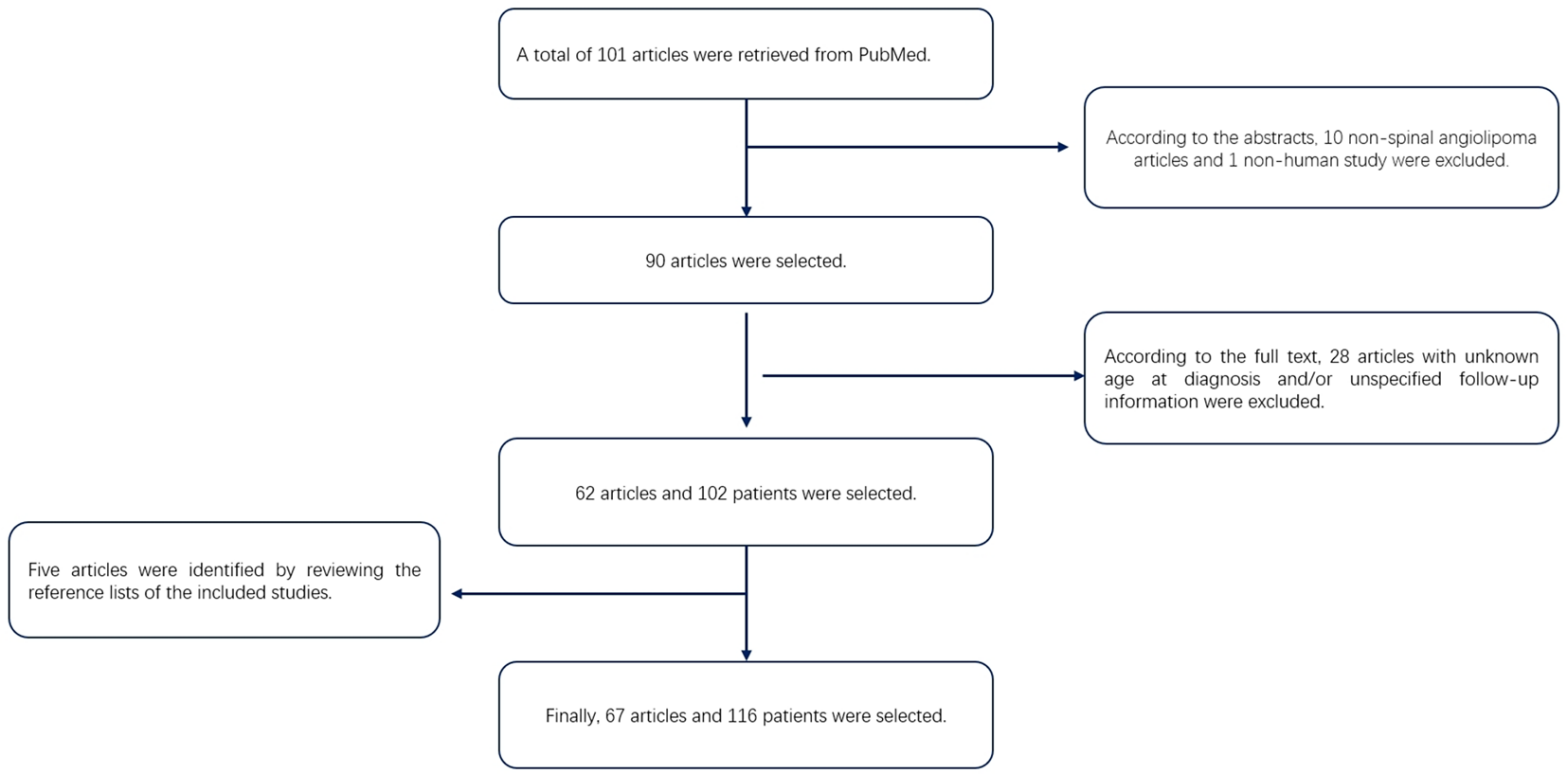
Flowchart of patient selection from the literature review.

The search of PubMed yielded 101 relevant English-language reports; 67 reports^2,4,6–70^ and 116 patients diagnosed with spinal epidural angiolipoma were ultimately included in this study. The flow chart of the study selection process is shown in Fig. 3. Essential information from the literature review is presented in Supplementary Table 1. We pooled patient data from our institution and the studies retrieved from PubMed and then analyzed their clinical characteristics and treatment. The findings of this analysis are presented in Table 1. In this group, 63.0% (75/119) of the lesions were located at the thoracic level. 31.9% (38/119) of these lesions affected two vertebral bodies, while a total of 75.6% (90/119) involved 2–4 vertebral bodies. The most prevalent symptoms experienced by patients were back pain, paraparesis, and numbness in the legs. Surgery was the primary treatment option for most patients, and complete tumor resection was achieved in most patients. Given that the ethnicity of patients was rarely mentioned in the literature, we collected the geographical locations of the hospitals where the patients in the studies were treated (Supplementary Table 1). We found that 64.7% (77/119) of the cases were reported by Asian institutions, and 29.4% (35/119) of the cases were reported by European and North American institutions. Only three cases were reported by institutions from Africa, and only one individual among the 119 patients was explicitly identified as being of African descent.

**Table 1:** Clinical characteristics and treatments of the patients from the literature review and our institution.

Variables	Number	Percent
Tumor location		
Cervicothoracic	7	5.9
Thoracic	75	63.0
Thoracolumbar	3	2.5
Lumbar	29	24.4
Lumbosacral	4	3.4
Sacral	1	0.8
Number of vertebral bodies involved		
1	12	10.1
2	38	31.9
3	33	27.7
4	19	16.0
5	8	6.7
6	2	1.7
7	4	3.4
8	1	0.8
9	2	1.7
First symptom		
Paraparesis	37	31.1
Numbness in legs	26	21.8
Back pain	48	40.3
Sciatalgia	8	6.7
Surgery		
Total resection	105	88.2
Partial resection	10	8.4
Unspecified	4	3.4

### Population-based analysis using the SEER database and descriptive analysis

**Figure 4:**
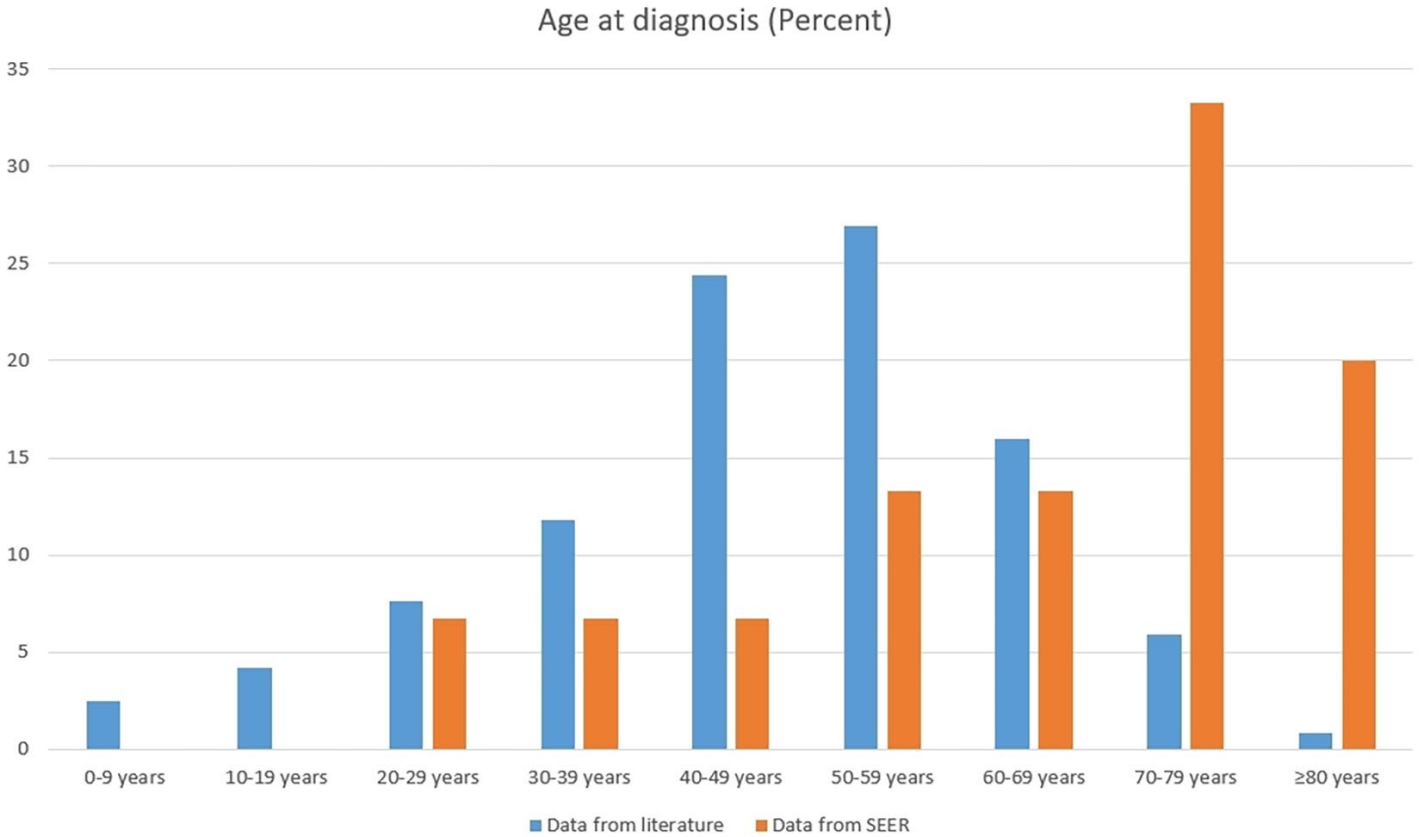
Distribution of patients from the literature and the SEER database by age at diagnosis.

We indexed 28,024 patients with primary spinal cord tumors from the SEER database, and only 15 patients were confirmed to have spinal angiolipoma, accounting for 0.05% (15/28,024) of all primary spinal cord tumors. Of these 15 patients, one was identified as Asian or Pacific Islander, while the remaining 14 were white. The results of the descriptive analysis of the demographic characteristics of the patients from the literature (including the patients from our institution) and the patients from the SEER database are shown in Table 2. We found that the male:female ratio in the published studies was 1:1.4 (50:69), while the 15 patients retrieved from the SEER database included 10 males and 5 females, yielding a male:female ratio of 2:1. The median age at diagnosis for the patients from the literature was 49 years old, with a median follow-up duration of 24 months. Conversely, for the patients obtained from the SEER database, the median age at diagnosis was greater (71 years), and the median follow-up duration was longer (72 months) (Table 2). The age distribution of these patients is illustrated in Fig. 4. Notably, the patients from the literature exhibited a peak incidence in the fifth decade of life. However, this peak was not observed among the data derived from the SEER database.

**Table 2:** Results of descriptive analysis investigating the demographic characteristics of patients from the literature and patients from the SEER database.

Variables	Data from the literature		Data from the SEER	
	Number	Percent	Number	Percent
Sex				
Female	69	58.0	5	
Male	50	42.0	10	
Age at diagnosis (years)				
Mean ± SD	48.00 ± 16.297		64.33 ± 17.731	
Median	49		71	
Range	1–81		28–85	
0–19	8	6.7	0	0
20–39	23	19.3	2	13.3
40–59	61	51.3	3	20.0
≥ 60	27	22.7	10	66.7
*Race*				
White	–	–	14	93.3
Asian or Pacific Islander	–	–	1	6.7
Follow up (months)				
Mean ± SD	29.05 ± 26.979		84.73 ± 55.116	
Median	24		72

## Discussion

The first case report of spinal angiolipoma was published by Berembruch in 1890^71^, and the first description was provided by Liebscher in 1901^72^. In 1960, Howard and Helwig defined angiolipoma as an anatomopathological entity containing mature fat cells and proliferating vessels^73^, and Haddad distinguished angiolipomas from lipomas in 1986^31^. Spinal angiolipomas account for 0.14–1.2% of all spinal tumors and approximately 2–3% of all extradural spinal tumors^3^. Pagni et al.^4^ reported three patients diagnosed with spinal epidural angiolipoma within a span of less than 2 years in a specific geographical area. This discovery raised the question of whether this tumor is genuinely rare or simply underreported. Herein, we observed that spinal epidural angiolipomas accounted for 0.05% of all primary spinal cord tumors based on data from the SEER database. Additionally, we found only 15 patients with spinal cord angiolipoma in the SEER 22 database, and we identified only three patients within our institution over a 10-year period. These findings support the theory that this tumor is indeed exceptionally rare, potentially even rarer than previously reported.

The etiology of spinal angiolipomas has yet to be fully elucidated. Bucy and Ehni characterized angiolipomas as congenital malformations^18,74^, while other authors have suggested that although these lesions should be classified as congenital tumors, they can expand in response to injury and/or inflammation^18,75–77^. Cases of spinal angiolipoma in pregnant women have been reported^11,21,28^. Tsutsumi et al.^21^ suggested that pregnant patients with spinal angiolipomas are at risk of developing acute epidural hemorrhage due to increased paraspinal venous pressure.

Spinal angiolipomas frequently manifest at the thoracic level^29,64^ and typically present with back pain and symptoms of progressive spinal cord compression^29^. Spinal epidural angiolipomas often present with non-specific clinical symptoms similar to other benign space-occupying spinal lesions, making it challenging to diagnose angiolipomas based on symptoms alone definitively^70^. A histopathological examination is crucial for confirming the diagnosis of spinal angiolipomas. Histologically, they consist of mature adipocytes and blood vessels, with characteristics resembling capillary hemangiomas, cavernous hemangiomas, or arteriovenous malformations^78^. Spinal angiolipomas are classified into two subtypes: Non-infiltrating and infiltrating^75^. The majority of spinal extradural angiolipomas are benign and non-infiltrative. The infiltrative type is generally located in the anterior epidural space and tends to invade the surrounding tissues, which could be mistaken for an aggressive tumor^17^. According to our literature review results, the infiltrative type had seldom been reported, and all three cases from our institution were non-infiltrating spinal angiolipomas.

MRI is one of the best imaging modalities for assessing spinal angiolipomas^79^. The unique combination of adipose tissue and vascular structures contributes to the distinctive appearance of spinal angiolipomas on MRI scans^80^. The adipose tissue appears hyperintense on both T1- and T2-weighted images but hypointense on fat-suppressed sequences. Meanwhile, the vascular component appears hypointense on T1-weighted images, hyperintense on T2-weighted images, and exhibits marked enhancement on post-contrast images^51^. In addition, it is essential to differentiate intradural angiolipoma from lipoma and cavernous hemangioma. A lipoma exhibits high signal intensity on T1WI and T2WI, low signal on lipoma pressure sequence, and no enhancement on contrast-enhanced scans. In contrast, a cavernous hemangioma displays low signal intensity on T1WI, high signal intensity on T2WI, no significant fat suppression, and marked enhancement on contrast-enhanced scans^51,79,81^.

Surgical resection is the gold standard for the treatment of spinal epidural angiolipomas, with the aim of complete tumor removal^59^. Some authors oppose adjuvant radiation, even in cases of partial resection^19,59^. Our literature review also showed that total resection was achieved in 88.2% (105/119) of patients, the use of adjuvant radiation is sparse. Si et al. performed a retrospective study of spinal angiolipomas, dividing patients into IA and IB categories. Type IB tumors are characterized by lipomatosis in their upper and/or lower segments, in contrast to the conventional type IA. The authors suggest that for patients with Type IB tumors, it is crucial to remove the angiolipoma and as much of the surrounding fat as possible. If complete removal of lipomatosis is unachievable, dietary therapy aimed at weight loss may be considered^80^. The median follow-up for patients reported in the literature review is 24 months. After surgery, 37% of patients showed improvement and 61.3% showed recovery. Only two patients had a stable or unchanged condition. Given the favorable prognosis for patients with spinal angiolipoma, in which cases of death or recurrence are rarely reported, we have not included a survival analysis in this study. At the same time, the relatively small numbers of patients from our institution limits the provision of new insights into the surgical dimensions of their treatment.

There is some general consensus regarding the demographic and clinical characteristics of patients with spinal epidural angiolipomas. For example, angiolipomas are more common in females than in males^18,19,28^ and are most commonly diagnosed during the fifth decade of life. Although our findings are largely consistent with existing research, we have also reported some results that are different from those of previous studies. Previous studies have predominantly indicated a greater prevalence of spinal epidural angiolipomas among women than among men^11,18,19,28^. However, these findings were not corroborated in retrospective analyses with larger sample sizes^64,81,82^. And the data from the SEER database did not reveal a predominance of female patients relative to male patients. Specifically, the male:female ratio of the patients in the SEER database was 2:1. Moreover, a peak incidence in the fifth decade of life was not observed in the data derived from the SEER database. Therefore, it is necessary to conduct an extensive retrospective analysis to examine discrepancies in the prevalence of this disease across various age groups and sexes.

Few studies have examined the potential race-based differences in the prevalence of spinal epidural angiolipoma. While the current literature review yielded a greater volume of cases from Asia, the data extracted from the SEER database did not confirm the predominance of Asian ethnicity among patients with spinal epidural angiolipoma. Asia is the most populous continent, which could have influenced these findings. In addition, retrospective analyses with the largest patient cohorts were conducted in Asia. Wang et al.^64^ conducted a retrospective analysis of the medical records of 20 patients to examine the clinical characteristics and surgical outcomes of spinal epidural angiolipomas. Researchers from Italy have posed the question "Spinal Epidural Angiolipoma: Rare or Unreported?"^4^ Interestingly, there are very few reports of spinal epidural angiolipomas in Africa, despite Africa being the world’s second most populous continent. Thus, we hypothesize that the incidence rate of spinal epidural angiolipoma is potentially lower among individuals of African descent than among those of Caucasian or Asian descent. However, this assumption needs to be further validated via exhaustive and large-scale retrospective studies. Although we got some results differing from previous knowledge about spinal epidural angiolipoma, we attempted to verify them through population-based analysis according to the SEER database. However, we were only able to index 15 cases from the SEER 22 registries database, which is currently the most comprehensive publicly available resource. A more extensive population-based analysis, using databases such as The Central Brain Tumor Registry of the United States (CBTRUS) may provide a more accurate picture of the true situation of this disease. At the same time, genetic and molecular analysis of spinal epidural angiolipomas might significantly improve our understanding of the disease.

This study has several limitations that must be considered. First, the follow-up MRI scans for the second and third patients from our institution were performed at an external local hospital, so we were unable to obtain the postoperative images. Second, we cannot definitively exclude the possibility of duplicate cases between the literature review and the entries in the SEER database. However, we effectively reduced the potential impact of patient duplication by consciously refraining from completely merging patients from these two sources. Despite these limitations, we believe that our research can assist clinicians in obtaining a deeper understanding of this rare tumor and provide guidance for future research endeavors.

## Conclusion

We presented three cases of spinal epidural angiolipoma and supplemented our findings with a literature review and population-based analysis according to the SEER database for the United States population. Our investigation indicated that spinal epidural angiolipoma is exceptionally rare, with some demographic features differing from those reported in previous studies. We believe that our research will enhance clinicians’ comprehension of this uncommon tumor.

### Supplementary Information

Supplementary Table 1.

## Data Availability

The datasets used and/or analyzed during the current study available from the corresponding author on reasonable request.
