# Supplementary material for: Demographic and clinical data of patients with spinal epidural angiolipomas
Source: Sci Rep. 2024 Jul 29;14:17473. doi: 10.1038/s41598-024-67584-8 (PMC11289124; doi:10.1038/s41598-024-67584-8)
Supplement: Supplementary file 1 — Supplementary Table 1. [file 41598_2024_67584_MOESM1_ESM.docx]

**Supplementary Table 1:** The essential information from the literature review.

| Authors | Country | Age/Sex | Tumor location | Infiltrating | Extent of resection | Radiotherapy | Complication | Outcome | Follow-up |
| --- | --- | --- | --- | --- | --- | --- | --- | --- | --- |
| Schiffer (1980)^30^ | Israel | 48/F | T10-L1 | Infiltrating | Total resection | No | No | Recovery | 20 months |
| Miki (1981)^6^ | Japan | 46/F | T3-5 |  | Total resection | No | No | Recovery without recurrence | 11 months |
| Haddad (1986)^31^ | Lebanon | 34/M | T8-10 | Infiltrating | Total resection | No | No | Recovery | 36 months |
| Haddad (1986)^31^ | Lebanon | 22/M | T8 |  | Total resection | No | No | Recovery | 12 months |
| Poon (1988)^32^ | United States (U.S.) | 65/F | T8-9 |  | Unspecified | No | No | Recovery | 36 months |
| Kuroda (1990)^8^ | Japan | 73/F | T4 | Infiltrating | Total resection | No | No | Recovery without recurrence | 24 months |
| Anson (1990)^7^ | U.S. | 58/F | T2-6 |  | Partial resection | Yes | No | Recovery | 10 weeks |
| Anson (1990)^7^ | U.S. | 65/F | T1-9 |  | Total resection | No | Dyskinesia | Improvement | 2 weeks |
| Rubin (1992)^9^ | Israel | 58/F | T8-10 |  | Total resection | No | No | Recovery | 4 months |
| Pagni (1992)^4^ | Italy | 56/F | L3 | Infiltrating | Total resection | No | No | Recovery | 2 months |
| Pagni (1992)^4^ | Italy | 59/F | L4-5 |  | Partial resection | No | No | Recovery | 3 months |
| Preul (1993)^11^ | Canada | 58/M | T3 | Infiltrating | Total resection | No | No | Recovery | 10 months |
| Preul (1993)^11^ | Canada | 45/F | T7-11 |  | Total resection | No | No | Improvement | 24 months |
| Preul (1993)^11^ | Canada | 36/F | T5-8 |  | Partial resection | No | No | Recovery | 30 months |
| Michilli (1993)^10^ | Germany | 12/M | T6-12 |  | Total resection | No | No | Recovery | 3 weeks |
| Shibata (1993)^12^ | Japan | 38/F | T4-6 |  | Total resection | No | No | Recovery without recurrence | 24 months |
| Yamashita (1993)^13^ | Japan | 57/M | T3-9 |  | Total resection | No | No | Recovery | 36 months |
| Preul (1993)^62^ | Canada | 68/F | T5-6 |  | Total resection | No | Mild spasms | Improvement | 6 months |
| Bouramas (1995)^33^ | Greece | 27/F | T4-7 |  | Total resection | No | No | Improvement | 6 months |
| Krishnan (1996)^14^ | New Zealand | 55/F | T7 |  | Total resection | No | No | Recovery | 10 days |
| Trabulo (1996)^66^ | Portugal | 26/F | T6-8 |  | Total resection | No | No | Recovery | 24 months |
| Trabulo (1996)^66^ | Portugal | 72/M | T5-6 | Infiltrating | Total resection | No | No | Recovery | 27 months |
| Oge (1999)^61^ | Turkey | 72/M | T3 |  | Total resection | No | No | Recovery | 24 months |
| Turgut (1999)^67^ | Turkey | 54/F | T4-9 |  | Total resection | No | No | Recovery | 9 months |
| AL-ANAZI (2000)^68^ | Saudi Arabia | 38/F | C5-9 |  | Total resection | No | No | Recovery | 12 months |
| Fourney (2001)^15^ | Canada | 46/F | T6-8 |  | Total resection | No | No | Recovery without recurrence | 24 months |
| Gelabert-González (2002)^16^ | Spain | 4/M | T2-5 |  | Total resection | No | No | Recovery | 17 days |
| Leu (2003)^17^ | China | 81/M | T3-5 | Infiltrating | Partial resection | No | No | Improvement | 2 months |
| doSouto (2003)^34^ | Brazil | 46/F | L4-5 | Infiltrating | Total resection | No | No | Recovery without recurrence | 18 months |
| Samdani (2004)^2^ | U.S. | 49/F | T6-8 | Infiltrating | Total resection | No | No | Recovery without recurrence | 6 months |
| Rabin (2004)^35^ | Canada | 47/M | T9 |  | Partial resection | No | No | Recovery | 12 months |
| Rocchi (2004)^36^ | Italy | 60/M | L3-4 |  | Total resection | No | No | Recovery | 24 months |
| Petrella (2005)^18^ | Italy | 16/M | T4-8 |  | Total resection | No | No | Recovery without recurrence | 6 months |
| Dogan (2006)^37^ | Turkey | 50/F | L4-5 | Infiltrating | Total resection | No | No | Recovery without recurrence | 12 months |
| Dogan (2006)^37^ | Turkey | 36/M | L1-2 |  | Total resection | No | No | Recovery without recurrence | 12 months |
| Konya (2006)^38^ | Turkey | 60/F | L5 |  | Total resection | No | No | Recovery without recurrence | 12 months |
| Akhaddar (2008)^39^ | Morocco | 47/M | T2-3 |  | Total resection | No | No | Recovery without recurrence | 24 months |
| Hungs (2008)^40^ | U.S. | 52/F | T2-5 |  | Total resection | No | No | Recovery | 22 months |
| Nanassis (2008)^41^ | Greece | 47/F | L2-3 |  | Total resection | No | No | Recovery without recurrence | 18 months |
| Yen (2008)^42^ | China | 71/M | T5-7 | Infiltrating | Partial resection | No | No | Recovery without progression | 18 months |
| Gelabert-González (2009)^19^ | Spain | 16/M | L5-S1 |  | Total resection | No | No | Recovery | 12 months |
| Gelabert-González (2009)^19^ | Spain | 45/F | L5-S1 |  | Total resection | No | No | Recovery | 6 months |
| Tsutsumi (2011)^21^ | Japan | 26/F | T3-4 |  | Total resection | No | No | Recovery | 12 months |
| Diyora (2011)^20^ | China | 20/M | T5-8 |  | Total resection | No | No | Recovery | 3 months |
| Ghanta (2012)^43^ | India | 56/M | T4-5 |  | Total resection | No | No | Recovery | 60 months |
| Han (2012)^44^ | China | 58/M | T4-5 | Infiltrating | Partial resection | No | No | Recovery without recurrence | 18 months |
| Fujiwara (2013)^22^ | Japan | 64/F | T5-8 |  | Total resection | No | No | Recovery without recurrence | 36 months |
| Fujiwara (2013)^22^ | Japan | 65/M | T5-8 |  | Total resection | No | No | Recovery without recurrence | 9 months |
| Nakao (2014)^23^ | Japan | 32/F | T1-6 |  | Total resection | No | No | Recovery without recurrence | 6 months |
| Ramdasi (2014)^45^ | India | 58/M | C7-T1 |  | Total resection | No | No | Recovery | 24 months |
| Eap (2015)^24^ | France | 22/M | C7-T3 |  | Total resection | No | No | Recovery | 3 months |
| Nadi (2015)^25^ | Canada | 50/F | T6-9 |  | Total resection | No | No | Recovery | 36 months |
| Benvenutti-Regato (2015)^46^ | Mexico | 65/F | T8-10 |  | Total resection | No | No | Recovery without recurrence | 6 months |
| Sandvik (2015)^47^ | Sweden | 1/M | C6-T7 |  | Total resection | No | No | Recovery without recurrence | 24 months |
| Mohammed (2016)^69^ | Sudan | 35/F | C5-8 |  | Total resection | No | No | Recovery | 12 months |
| Sim (2016)^48^ | Australia | 58/F | T2-6 | Infiltrating | Total resection | No | No | Improvement | 2 months |
| Ying (2017)^26^ | Korea | 63/M | L2-3 |  | Total resection | No | No | Recovery without recurrence | 12 months |
| Kang (2017)^49^ | China | 69/M | L2-3 |  | Total resection | No | No | Recovery | 3 months |
| Onishi (2017)^50^ | Japan | 35/F | T3-5 |  | Total resection | No | No | Recovery without recurrence | 24 months |
| Wang (2017)^51^ | China | 25/F | L3-4 |  | Unspecified | No | No | Recovery | 2 months |
| Wang (2017)^51^ | China | 77/F | T2-4 |  | Unspecified | No | No | Improvement | 2 months |
| Wang (2017)^51^ | China | 45/F | T4-6 |  | Total resection | No | No | Recovery | 10 months |
| Lacour (2018)^27^ | France | 17/M | T9-10 |  | Total resection | No | No | Improvement | 2 months |
| Horiuchi (2018)^52^ | Japan | 49/F | T5-8 |  | Total resection | No | No | Recovery without recurrence | 12 months |
| Rkhami (2018)^53^ | Tunisia | 65/F | T7-10 |  | Total resection | No | Urinary leaks | Improvement | 60 months |
| Maduri (2019)^28^ | Switzerland | 29/F | T5-9 |  | Total resection | No | No | Recovery without recurrence | 6 months |
| Iampreechakul (2020)^29^ | Thailand | 55/F | S3-4 |  | Unspecified | No | No | Recovery without recurrence | 24 months |
| Apostolakis (2020)^54^ | Greece | 35/M | T7-9 |  | Total resection | No | No | Recovery without recurrence | 6 months |
| Apostolakis (2020)^54^ | Greece | 46/M | T9-L3 |  | Partial resection | No | No | Recovery without recurrence | 7 months |
| Cheng (2020)^55^ | China | 54/M | T3 |  | Total resection | No | No | Recovery without recurrence | 60 months |
| Ikpeze (2020)^56^ | U.S. | 39/F | L1-2 |  | Total resection | No | No | Improvement | 48 months |
| Jaiswal (2020)^57^ | Germany | 1.5/F | T5-9 |  | Total resection | No | Uroschesis | Improvement | 3 months |
| Wang (2020)^58^ | China | 47/F | L1-2 |  | Total resection | No | No | Improvement | 12 months |
| Wang (2020)^58^ | China | 61/M | T5-7 |  | Total resection | No | No | Recovery without recurrence | 12 months |
| Albano (2020)^65^ | U.S. | 31/M | T4-5 |  | Total resection | No | No | Improvement | 2 days |
| Yang (2021)^63^ | China | 58/F | L4-5 |  | Total resection | No | Unspecified | Recovery | 84 months |
| Yang (2021)^63^ | China | 51/M | T4-8 |  | Total resection | No | Unspecified | Improvement | 80 months |
| Yang (2021)^63^ | China | 50/M | T5-6 |  | Total resection | No | Unspecified | Improvement | 74 months |
| Yang (2021)^63^ | China | 49/F | T8-11 |  | Total resection | No | Unspecified | Stable | 71 months |
| Yang (2021)^63^ | China | 69/F | T4-6 |  | Partial resection | No | Unspecified | Improvement | 63 months |
| Yang (2021)^63^ | China | 61/M | T2-3 |  | Total resection | No | Unspecified | Improvement | 62 months |
| Yang (2021)^63^ | China | 25/M | C7-T1 |  | Total resection | No | Unspecified | Recovery | 25 months |
| Yang (2021)^63^ | China | 60/F | L2-3 |  | Total resection | No | Unspecified | Recovery | 18 months |
| Yang (2021)^63^ | China | 64/F | T7 |  | Total resection | No | Unspecified | Improvement | 17 months |
| Yang (2021)^63^ | China | 49/M | L3-4 |  | Total resection | No | Unspecified | Recovery | 14 months |
| Somrani (2022)^59^ | Tunisia | 54/F | L5-S1 |  | Total resection | No | No | Recovery without recurrence | 24 months |
| Zhang (2022)^70^ | China | 67/M | L5 | Infiltrating | Total resection | No | No | Improvement | 54 months |
| Zhang (2022)^70^ | China | 47/F | T6-8 |  | Total resection | No | No | Recovery | 50 months |
| Zhang (2022)^70^ | China | 45/F | T6-7 |  | Total resection | No | No | Improvement | 36 months |
| Zhang (2022)^70^ | China | 37/F | T11-12 |  | Total resection | No | No | Recovery | 34 months |
| Zhang (2022)^70^ | China | 36/M | T8-10 | Infiltrating | Total resection | No | No | Improvement | 33 months |
| Zhang (2022)^70^ | China | 54/F | T4-6 |  | Total resection | No | No | Recovery | 26 months |
| Zhang (2022)^70^ | China | 12/M | C4-6 |  | Total resection | No | No | Improvement | 25 months |
| Zhang (2022)^70^ | China | 48/M | L1-3 |  | Total resection | No | No | Improvement | 22 months |
| Zhang (2022)^70^ | China | 73/F | L3-5 |  | Total resection | No | No | Improvement | 21 months |
| King (2023)^60^ | U.S. | 44/M | T2-3 |  | Total resection | No | Constipation | Improvement | 9 days |
| Wang (2023)^64^ | China | 51/F | T4-7 |  | Total resection | No | Unspecified | Improvement | 56.9 months (mean) |
| Wang (2023)^64^ | China | 74/F | T4-7 |  | Total resection | No | Unspecified | Improvement | 56.9 months (mean) |
| Wang (2023)^64^ | China | 50/M | T7 |  | Total resection | No | Unspecified | Improvement | 56.9 months (mean) |
| Wang (2023)^64^ | China | 54/M | T5-7 |  | Total resection | No | Unspecified | Improvement | 56.9 months (mean) |
| Wang (2023)^64^ | China | 60/M | T2-10 |  | Partial resection | No | Unspecified | no change | 56.9 months (mean) |
| Wang (2023)^64^ | China | 58/F | T8-10 |  | Total resection | No | Unspecified | Improvement | 56.9 months (mean) |
| Wang (2023)^64^ | China | 64/M | L2-3 |  | Total resection | No | Unspecified | Improvement | 56.9 months (mean) |
| Wang (2023)^64^ | China | 52/F | T4-7 |  | Total resection | No | Unspecified | Improvement | 56.9 months (mean) |
| Wang (2023)^64^ | China | 47/F | T3-8 |  | Total resection | No | Unspecified | Improvement | 56.9 months (mean) |
| Wang (2023)^64^ | China | 55/F | T6-8 |  | Total resection | No | Unspecified | Improvement | 56.9 months (mean) |
| Wang (2023)^64^ | China | 38/M | L4-S1 |  | Total resection | No | Unspecified | Improvement | 56.9 months (mean) |
| Wang (2023)^64^ | China | 48/F | L2-3 |  | Total resection | No | Unspecified | Improvement | 56.9 months (mean) |
| Wang (2023)^64^ | China | 46/F | T2-4 |  | Total resection | No | Unspecified | Improvement | 56.9 months (mean) |
| Wang (2023)^64^ | China | 46/M | L2-3 |  | Total resection | No | Unspecified | Improvement | 56.9 months (mean) |
| Wang (2023)^64^ | China | 51/M | T1-3 |  | Total resection | No | Unspecified | Improvement | 56.9 months (mean) |
| Wang (2023)^64^ | China | 55/F | T12-L1 |  | Total resection | No | Unspecified | Improvement | 56.9 months (mean) |
| Wang (2023)^64^ | China | 46/F | L2-4 |  | Total resection | No | Unspecified | Improvement | 56.9 months (mean) |
| Wang (2023)^64^ | China | 44/F | T8-10 |  | Total resection | No | Unspecified | Improvement | 56.9 months (mean) |
| Wang (2023)^64^ | China | 48/M | T11-12 |  | Total resection | No | Unspecified | Improvement | 56.9 months (mean) |
| Wang (2023)^64^ | China | 55/M | T5-7 |  | Total resection | No | Unspecified | Improvement | 56.9 months (mean) |
| Our case 1 | China | 52/F | T4-6 |  | Total resection | No | No | Recovery | 29 months |
| Our case 2 | China | 49/F | L1-3 |  | Total resection | No | No | Recovery | 14 days |
| Our case 3 | China | 47/F | T2-5 |  | Total resection | No | No | Recovery | 10 years |
